# Human parechovirus infection in central nervous system related diseases and sepsis in children in Shanghai, China

**DOI:** 10.1186/1753-6561-5-S6-P47

**Published:** 2011-06-29

**Authors:** J Xu, H Zhong, Y Yang

**Affiliations:** 1 Pediatric Institute, Children’s Hospital, Shanghai, China

## Introduction / objectives

Human parechovirus (HPeVs) are prevalent in young children and have been associated with mild gastroenteritis, respiratory diseases, meningitis/encephalitis, myocarditis and sepsis. In this study we determined the relative importance of HPeV involoved in the development of central nervous system-associated disease and sepsis.

## Methods

A total of 1225 enterovirus-negative specimens including 674 cerebrospinal fluid (CSF), 550 blood samples and one ascitic fluid sample from 1131 children <14 years of age obtained in the year of 2008 to 2010 were screened for HPeV by nested PCR. All positive samples were genotyped by sequencing of VP3/VP1genes.

## Results

HPeV was detected in 96 samples from 92 (8.1%) of the children. Yearly prevalence of HPeV in CSF and blood varied remarkably: 1.3% (2/153) in 2008, 7.4% (27/328) in 2009 and 10.0% (63/631) in 2010. In 2008, HPeV infections were observed only in December. HPeV was detected mainly in autumn and winter, with the peak in December (18.8%) in the year of 2009. In 2010, HPeV could be found throughout the year with the highest prevalence in January (24.2%). HPeV infections were only found in infants less than 1 year old in 2008. However, HPeV infections can be detected in all age groups of children during the year of 2009 and 2010. Of all the 49 strains genotyped successfully, 48 were identified to be HPeV1 and the other one was HPeV 3 which was detected in 2010. Of the 92 Children with HPeV infections, 86 had clinical symptoms of central nervous infections, 33 were diagnosed to be sepsis. 4 patients were dead and 2 refused further treatment because of severe meningitis.

## Conclusion

HPeV was a significant cause of central nervous system infections and sepsis in children in Shanghai, China. HPeV 1 was identified to be the most predominant type during 2008 to 2010**.**

## Disclosure of interest

None declared.

